# Novel peripheral blood parameters as predictors of neoadjuvant chemotherapy response in breast cancer

**DOI:** 10.3389/fsurg.2022.1004687

**Published:** 2022-11-04

**Authors:** Gaohua Yang, Pengju Liu, Longtian Zheng, Jianfeng Zeng

**Affiliations:** Department of General Surgery, The Second Affiliated Hospital of Fujian Medical University, Fujian Province, Quanzhou, China

**Keywords:** breast cancer, neoadjuvant chemotherapy, neutrophil-to-lymphocyte ratio, platelet-to-lymphocyte ratio, systemic immune severity index

## Abstract

The neutrophil-to-lymphocyte ratio (NLR), platelet-to-lymphocyte ratio (PLR), systemic immune severity index (SII), and prognostic nutritional index (PNI) are associated with the prognosis of gastric, lung, and breast cancers. However, the predictive value of pathological complete response (pCR) rates in patients with breast cancer treated with neoadjuvant chemotherapy (NAC) remains unclear. This retrospective study explored the correlation between each index and the efficacy of neoadjuvant chemotherapy in patients with breast cancer and assessed the relationship between changes before and after neoadjuvant chemotherapy. We enrolled 95 patients with locally advanced breast cancer who received neoadjuvant therapy for breast cancer at the Second Affiliated Hospital of Fujian Medical University from April 2020 to April 2022. Based on postoperative pathology, patients were divided into pCR and non-pCR groups. Between-group differences and efficacy prediction ability of NLR, PLR, SII, and PNI were analyzed. Patient characteristics and changes in NLR, PLR, SII, and PNI before and after neoadjuvant chemotherapy (NAC) were compared between groups. Patients were divided into two groups according to the optimal diagnostic thresholds of the SII before treatment. Between-group differences in terms of neoadjuvant therapy efficacy and patient characteristics were evaluated. The pCR exhibited significantly lower ER (*χ*^2 ^= 10.227, *P* = 0.001), PR (*χ*^2 ^= 3.568, *P* = 0.049), pretreatment NLR (*χ*^2 ^= 24.930, *P* < 0.001), pretreatment PLR (*χ*^2 ^= 22.208, *P* < 0.001), pretreatment SII (*χ*^2 ^= 26.329, *P* < 0.001), and post-treatment PNI (*P* = 0.032), but higher HER-2 (*χ*^2 ^= 7.282, *P* = 0.007) and ΔNLR (*P* = 0.015) than the non-pCR group. ROC curve analysis revealed that the areas under the curve (AUC) of pretreatment SII, NLR, and PLR for predicting pCR of NAC for breast cancer were 0.827, 0.827, and 0.810, respectively, indicating a higher predictive value for response to NAC in patients with breast cancer. According to the Youden index, the optimal cut-off value of SII pretreatment was 403.20. Significant differences in age (*χ*^2 ^= 6.539, *P* = 0.01), ER (*χ*^2 ^= 4.783, *P* = 0.029), and HER-2 (*χ*^2 ^= 4.712, *P* = 0.030) were observed between high and low-SII groups. In conclusion, pretreatment NLR, PLR, and SII can be used as predictors of pCR in patients with breast cancer receiving neoadjuvant chemotherapy. The predictive value of pretreatment SII is higher, and patients with low SII are more likely to achieve pCR.

## Introduction

Breast cancer is a malignant tumor with the highest morbidity and mortality among women worldwide. The prevalence of breast cancer is increasing and has become a critical public health issue. Progress in precision medicine has resulted in developments in treatment methods for breast cancer (1). At present, treatment of breast cancer predominantly involves surgery supplemented by systemic therapy and other individualized comprehensive treatment plans (2). However, for locally advanced tumors, such as tumor diameter > 5 cm, axillary lymph node metastasis, or poor molecular type (such as HER-2-positive or triple-negative); or for patients with a ratio of tumor size to breast volume that makes it difficult to preserve breasts, preoperative neoadjuvant drug therapy is often favored to achieve tumor down-staging and reduce recurrence rate in order to prolong patient survival (3). Previous studies have demonstrated that OS and RFS of patients receiving neoadjuvant chemotherapy (NAC) are closely related to the efficacy of neoadjuvant therapy. As patients who achieve pathological complete remission (pCR) with NAC typically have longer survival time, early prediction of efficacy in breast cancer is critical for individualized treatment (4).

The current preoperative NAC regimen for breast cancer is based on factors such as molecular classification and predominantly comprises a 6-cycle TEC or 8-cycle EC-T regimen, that is, taxane combined with anthracycline, For patients with HER-2-positive breast cancer, targeted drugs are often added, such as trastuzumab and pertuzumab. Each cycle consists of 21 days, and evaluations are performed once every three cycles. Surgery is performed after completing the entire NAC course, and the specific efficacy of neoadjuvant therapy is evaluated according to postoperative paraffin pathology. However, there is a paucity of relevant prediction methods in clinical practice for patients who are insensitive to neoadjuvant therapy and delayed treatment. Therefore, there is an urgent need to explore convenient and effective indicators to assist in the clinical evaluation of the efficacy of neoadjuvant therapy in patients with breast cancer. Given the ease of performing blood tests, assessment of peripheral blood-related indicators may hold considerable clinical value for predicting the efficacy of neoadjuvant therapy in patients with breast cancer.

Persistent subclinical inflammation is associated with various diseases, particularly senile diseases (5). Recent studies have reported that chronic inflammation is closely associated with the occurrence and development of cancer (6). Tumor recurrence and metastasis are associated with the biological behavior of tumors and inflammatory responses. In cancer, normal vascular endothelial cells regulate microenvironmental homeostasis that can limit tumor growth, invasion, and metastasis. In contrast, dysfunctional endothelial cells exposed to an inflammatory tumor microenvironment support cancer progression and metastasis (7). For example, the persistent presence of *Helicobacter pylori*-associated gastritis is inseparable from MALT lymphoma.

Neutrophils, platelets, lymphocytes, and albumin are key mediators of chronic inflammation. Based on studies examining different solid tumors, the poor prognosis of gastric, lung, and breast cancers is associated with increased neutrophil/lymphocyte ratio (NLR), platelet/lymphocyte ratio (PLR), systemic immune inflammatory index (SII), and prognostic nutritional index (PNI) (8). However, there is a paucity of studies on the relationship between these inflammatory indicators and the efficacy of neoadjuvant therapy in patients with breast cancer.

Therefore, this study aimed to analyze the relationship of NLR, PLR, SII, and PNI with the efficacy of NAC in patients with breast cancer. To this end, we explored the risk factors affecting the efficacy of NAC in patients with breast cancer and analyzed the relationship between NLR, PLR, SII, and PNI changes pre-NAC and post-NAC to derive pretreatment predictive indicators for individualized breast cancer treatment.

## Materials and methods

### Patients

A total of 95 patients with breast cancer who received preoperative neoadjuvant therapy at the Second Affiliated Hospital of Fujian Medical University between April 2020 and April 2022 were selected as research participants. We extracted detailed treatment information and clinical data from the medical records of all patients. The inclusion criteria were as follows: (1) pathologically diagnosed breast cancer based on ultrasound-guided needle biopsy; (2) completed a course of neoadjuvant therapy; and (3) no other distant organ metastasis. Exclusion criteria were as follows: (1) bilateral, multifocal, inflammatory breast cancer; (2) history of breast surgery or other cancers; (3) had not completed the full course of treatment; and (4) patients with immune-related diseases, chronic wasting diseases, and blood system diseases that affected blood testing.

### Clinical characteristics

Clinical data of the enrolled patients were collected, including age, NAC regimen, TNM stage, pathological type, histological grade, molecular typing, platelet count, neutrophil count, lymphocyte count, albumin count and efficacy evaluation. Evaluation of efficacy was predominantly based on whether the patient achieved pCR after NAC, i.e., no histological evidence of malignant tumor in the primary breast tumor and metastatic regional lymph nodes or carcinoma restricted to the *in situ* component based on the 2022 China Clinical Tumor Society (CSCO) guidelines for the diagnosis and treatment of breast cancer, which set the positive threshold of ER and PR immunohistochemical detection as 1% and immunohistochemical results of Her-2 (+++) as Her-2 positive. If Her-2 (++), FISH detection was included, and Her-2 status was determined based on FISH results. All patients were TNM-staged according to the American Joint Committee on Cancer guidelines.

### Methods

Based on the results of routine blood and biochemical tests pre- and post-NAC, platelet, neutrophil, lymphocyte, and albumin counts as well as NLR, PLR, SII, and PNI were calculated. NLR and PLR refer to the ratio of neutrophils to lymphocytes and platelets to lymphocytes, respectively. SII was calculated as follows: platelet count × neutrophil count/lymphocyte count, which reflects inflammation and immune system status. PNI was calculated as follows: albumin (g/L) + 5 × lymphocyte count (109/L). Patients were divided into PCR and non-PCR groups based on postoperative pathology. The efficacy prediction ability of NLR, PLR, SII, and PNI values before treatment and differences in clinicopathological characteristics between the two groups were analyzed. Dynamic changes in NLR, PLR, SII, and PNI were measured. The receiver operating characteristic (ROC) curve was used to determine the optimal cutoff value of SII, that is, the maximum point of the sum of sensitivity and specificity (Youden index), and divided into two groups (high and low) to compare the difference in efficacy of NAC and clinical outcomes between the two groups and between different pathological features.

### Statistical analysis

The database was established using Excel. Data were analyzed and graphed using SPSS 26.0 and GraphPad Prism 9.0 software. Quantitative data conforming to a normal distribution were expressed as x ± s, and two independent samples *t*-test was used for comparison between groups, the U test was used for the non-normal quantitative data, and qualitative data were expressed as the number of cases and percentages, and the comparison between groups was performed by chi-square test. The receiver operating characteristic (ROC) curve was drawn to analyze the predictive value of SII before treatment for pCR. The SII value corresponding to the maximum sum of sensitivity and specificity was the best cut-off value. *P* < 0.05 considered the difference to be statistically significant.

## Results

### Relationship between pCR grouping and clinical characteristics

A total of 95 patients with breast cancer were included in the study. Of patients, 26 achieved pCR after neoadjuvant therapy (pCR rate, 27.4%). Significant differences were observed in ER, PR, and HER-2 expression between the pCR and non-pCR groups (all *P* < 0.05). Patients with ER (−), PR (−), HER-2 (+++) exhibited higher pCR rates. No significant differences between the pCR and non-pCR groups were noted in age, tumor size, lymph node metastasis, and Ki-67 (*P* > 0.05).The data are summarized in (Table 1).

**Table 1:** Associations of clinicopathological characteristics with pCR in breast cancer patients.

Variables	Number	PCR	*n*-PCR	χ^2^	*P*-value
**Age**				1.422	0.233
≤50岁	49	16	33		
>50岁	46	10	36		
**TNM**				0.816	0.366
≤II	44	14	30		
>II	51	12	39		
**T**				0.460	0.498
≤5 cm	60	15	45		
>5 cm	35	11	24		
**N**				0.531	0.466
≤N1	64	19	45		
>N1	31	7	24		
**ki-67**				2.915	0.088
≤30%	35	6	29		
>30%	60	20	40		
**ER**				10.227	0.001
Negative	31	15	16		
Positive	64	11	53		
**PR**				3.568	0.049
Negative	40	15	25		
Positive	55	11	44		
**HER-2**				7.282	0.007
Low	47	7	40		
High	48	19	29		
**NLR**				24.930	0.000
>2.46	39	0	39		
<2.46	56	26	30		
**PLR**				22.208	0.000
>118.78	67	9	58		
<118.78	28	17	11		
**SII**				26.329	0.000
>403.20	74	11	63		
<403.20	21	15	6		

### Relationship of NLR, PLR, SII, and PNI with pCR

The 95 enrolled patients were divided into pCR and non-pCR groups. Dichotomous variables passed the *χ*^2^ test, and continuous variables passed the *t*-test. Pretreatment NLR, pretreatment PLR, and pretreatment SII were lower in the pCR group than in the non-pCR group. (*P* < 0.001). Post-treatment PNI and ΔNLR were lower in the non-pCR group (*P* < 0.05). In contrast, pretreatment PNI, post-treatment NLR, post-treatment PLR, SII, ΔPLR, ΔSII, and ΔPNI post-treatment were significantly different to those in the non-pCR group. No significant correlations were noted with the efficacy of NAC (all *P* > 0.05). None of the patients with a high pretreatment NLR achieved pCR. Patients with pretreatment PLR < 118.78 had a 4.5-fold higher rate of pCR compared to those with pretreatment PLR > 118.78. pCR rate was almost five times higher in patients with pretreatment SII < 403.20 than in those with pretreatment SII > 403.20. The data are summarized in (Table 2).

**Table 2:** The relationship between NLR, PLR, SII, PNI and pCR before treatment, after treatment and dynamic changes.

Variables	PCR	`X ± S	*T*-value	*P*-value
**Pre-NLR**	Yes	1.59±0.53	−6.278	<0.001
	No	2.81±1.36		
**Pre-PLR**	Yes	113.46±29.53	−5.747	<0.001
	No	168.01±62.45		
**Pre-SII**	Yes	409.34±170.62	−5.546	<0.001
	No	791.64±500.67		
**Pre-PNI**	Yes	55.74±4.28	0.678	0.500
	No	55.02±4.73		
**Post-NLR**	Yes	2.06±1.08	−1.136	0.259
	No	2.42±1.49		
**Post-PLR**	Yes	172.50±95.87	−1.633	0.106
	No	208.87±97.14		
**Post-SII**	Yes	482.81±381.08	−1.616	0.110
	No	646.90±461.60		
**Post-PNI**	Yes	52.01±3.80	2.173	0.032
	No	49.95±4.24		
**ΔNLR**	Yes	0.47±1.31	2.478	0.015
	No	−0.39±1.58		
**ΔPLR**	Yes	59.04±87.56	0.852	0.396
	No	40.85±94.62		
**ΔSII**	Yes	73.47±437.56	1.724	0.088
	No	−144.74±585.86		
**ΔPNI**	Yes	−3.73±4.74	1.688	0.095
	No	−5.08±2.84		

### Predictive value of preoperative NLR, PLR, SII, and PNI for pCR in breast cancer

**Figure 1:**
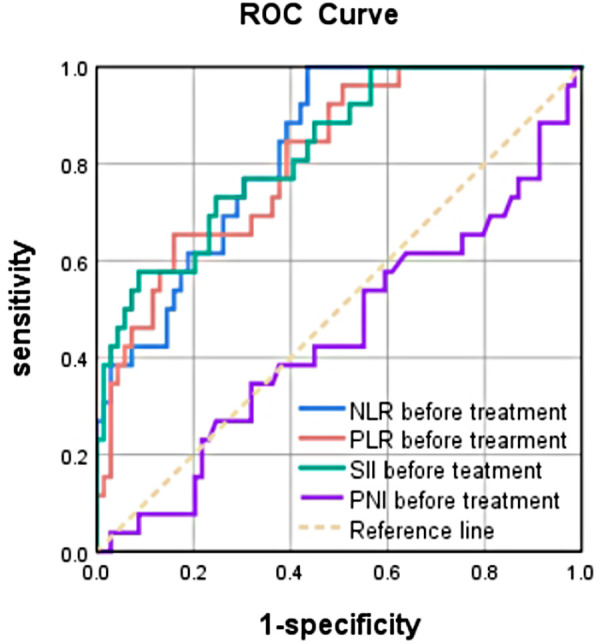
Predictive ability of the SII,NLR, PLR and PNI presented by ROC curves.

The area under the curve (AUC) of the ROC curve of NLR, PLR, SII, and PNI was used to assess the ability of preoperative NLR, PLR, SII, and PNI to predict pCR in patients with breast cancer. The AUC, best cut-off value, sensitivity, and specificity of NLR were 0.827 (95% CI: 0.744–0.910, *P* < 0.001), 2.46, 100%, and 56.5%, respectively. The AUC, best cut-off value, sensitivity, and specificity of PLR were 0.810 (95% CI: 0.718–0.901), *P* < 0.001), 118.78, 65.4%, and 84.1%, respectively. The AUC, best cut-off value, sensitivity, and specificity of SII were 0.827 (95% CI: 0.737–0.916, *P* < 0.001), 403.20, 57.7%, and 91.3%, respectively. The AUC of PNI was 0.444 (95% CI: 0.309–0.579, *P* > 0.05), indicating that NLR, PLR, and SII had good predictive values. Of these indices, SII had the largest AUC, and pretreatment PNI did not have significant predictive value. This suggested that pretreatment SII had higher predictive value for the efficacy of NAC in patients with breast cancer.The data are summarized in (Figure 1).

### Relationship between pretreatment SII grouping and clinical characteristics

Based on the optimal cut-off value of the ROC curve, patients were divided into groups with pretreatment SII <403.20 or >403.20. Significant between-group differences were observed in age, ER, and HER-2 status. Patients in the low SII group were predominantly aged ≤50 years, ER (−), and HER-2 (+++), while those in the high SII group were aged >50 years, with low expression of ER (+) and HER-2. Other indicators, such as tumor size, TNM stage, lymph node metastasis, and PR, were not significantly correlated with the high and low SII groups.The data are summarized in (Table 3).

**Table 3:** Relationships between SII and clinicopathological characteristics.

Variables	Number	SII<403.20	SII>403.20	*X*^2^值	*p*值
**Age**				6.539	0.01
≤50岁	49	16	33		
>50岁	46	5	41		
**TNM**				0.130	0.719
≤II	44	9	35		
>II	51	12	39		
**T**				0.143	0.706
≤5 cm	60	14	46		
>5 cm	35	7	28		
**N**				0.366	0.545
≤N1	64	13	51		
>N1	31	8	23		
**ki-67**				0.143	0,706
≤30%	35	7	28		
>30%	60	14	46		
**ER**				4.783	0.029
Negative	31	11	20		
Positive	64	10	54		
**PR**				2.501	0.114
Negative	40	12	28		
Positive	55	9	46		
**HER-2**				4.712	0.030
Low	47	6	41		
High	48	15	33		

## Discussion

Breast cancer has emerged as the most common cancer among women worldwide, and its mortality rate ranks first among cancer-related deaths in women, with a gradual upward trend (9). The specific mechanisms underlying the occurrence and development of breast cancer remain unclear. In recent years, the correlation between chronic inflammation and breast cancer has received widespread attention from the medical community (10). Numerous studies have demonstrated a relationship between chronic inflammation and poor prognosis in patients with breast cancer. The occurrence of breast cancer causes cancer cells to accumulate chemotactic inflammatory cells, while chronic inflammation-associated neutrophils regulate the tumor microenvironment (TME) through cytokines and cathepsins to promote cancer cell migration, invasion, and metastasis. A possible underlying mechanism is chronic inflammation-induced production of inflammatory mediators such as interleukin-6 (IL-6) (11). These effects promote angiogenesis in target organs, DNA damage, and gene mutation, which leads to cancer growth and metastasis. Further, nuclear transcription factor-*κ*B (NF-*κ*B), a key factor in inflammation and tumor cells, induces TNF-α and IL-6 chemotactic leukocytes to infiltrate the inflammation site (12). This may contribute to genetic mutations, thereby promoting tumorigenesis. In addition, activation of the STAT-3 and NF-*κ*B signaling pathways stimulates the expression of vascular endothelial growth factor (VEGF) and chemokines (CXC), induces epithelial-to-mesenchymal transition, and ultimately promotes tumor cell proliferation and growth (13). Therefore, we designed this study to investigate the relationship between breast cancer and chronic inflammation.

Neutrophils in the TME can be divided into two types: N1 and N2. N1 neutrophils exert anti-tumor properties and directly kill cancer cells through cytotoxicity, antibody-dependent cytotoxicity, and antigen presentation (14). N2-type neutrophils exert tumor-promoting properties by promoting cancer cell proliferation, pathological angiogenesis, and immune regulation. Queen et al. reported that neutrophils promote the expression of VEGF by releasing oncostatin M and binding to receptors on the cell membrane in breast cancer, thereby activating tyrosine kinase signaling pathways and transcriptional activators, ultimately promoting tumor invasion (15).

Platelets play a key role in the process of vascular injury repair (16). Platelets store and release vascular regulatory factors, such as VEGF, to increase vascular permeability, promote blood coagulation, and induce vascular endothelial cell migration, thereby promoting tumor angiogenesis (17). Indeed, platelets play an essential role in tumor invasion and metastasis. A retrospective analysis of 180 patients with breast cancer and 100 patients with normal breasts by Liu et al. revealed that the pCR rate of supraclavicular lymph nodes after NAC was 51.8%. In this regard, platelets have predictive value for the prognosis of patients with breast cancer and metastasis. Patients with high platelet counts have poorer prognosis compared to patients with low platelet counts, suggesting that platelet counts may be clinically useful for differentiating high-risk patients (18).

Peripheral blood lymphocytes reflect immune levels and overall nutritional status of the body. CD8 + T lymphocytes promote the anti-tumor ability of endogenous lymphocytes through type I immune responses and release perforin through the perforin-granzyme pathway (19). Natural killer cells can induce dendritic cells to aggregate within the TME by releasing chemokines and killing cancer cells or activating T lymphocytes to initiate specific immune responses through interferon (20). A retrospective analysis of the relationship between tumor-infiltrating lymphocytes and breast cancer by Tianen et al. revealed that regional infiltrating lymphocytes in the tumor could be used as a predictor of the efficacy of neoadjuvant therapy for breast cancer, and neoadjuvant therapy promoted immune infiltration of TILs in the tumor region of patients with breast cancer (21).

Previous studies have confirmed that NLR, PLR, and SII are associated with the prognosis of many malignant tumors, including colon, prostate, and breast cancers, and are closely related to the depth of tumor invasion and lymph node metastasis (22). Gulzade et al. demonstrated that a high NLR could be used as an independent predictor in the differential diagnosis of breast cancer from benign breast disease and could predict sentinel lymph node metastasis (23). Coh et al. analyzed pretreatment NLR in more than 2,000 patients with breast cancer and concluded that 5-year survival in the high NLR group was lower. Further, women with breast cancer in the high NLR group were younger, had larger tumor size, and had a higher risk of lymphatic and distant metastases (24). A retrospective study by Liu et al. reported that increased SII was associated with poorer OS in triple-negative breast cancer (HR = 2.91, *P* < 0.001) (25). Chen et al. used an SII of <602 × 10^9^/L as the optimal cut-off value and divided patients into high and low SII groups. DFS and OS of patients with breast cancer were higher in the low SII group than in the high SII group, and SII was not significantly associated with the side effects of NAC (26). Multivariate analysis in a propensity score-matched study on the prognostic value of preoperative SII in breast cancer initiated by Hua et al. revealed that SII independently predicted OS (*P* = 0.017) and DMFS (*P* = 0.007) (27). The prognosis of patients is closely associated with histological type, T stage, N stage, PR, HER2, and Ki67 of the tumor. Patients with breast cancer with a high initial SII value should thus receive early supplemental immunotherapy and anti-inflammatory treatment.Our study confirmed that pretreatment NLR, PLR, and SII could be used as predictors of the efficacy of locally advanced neoadjuvant therapy. Further, we observed that pCR was closely associated with ER, PR, and HER-2 but was not significantly related to tumor size or lymph node metastasis. A possible reason is potential bias due to the small sample size of this study. No significant differences were noted in post-treatment NLR, PLR, SII, ΔNLR, ΔPLR, and ΔSII between the two groups, possibly because the measured blood parameters were not sensitive enough to reflect the inflammatory state of the body due to the effects of bone marrow suppression after chemotherapy.

Serum albumin is a key indicator of the nutritional status of the body (28). In the pathological state of cancer, albumin consumption increases, and low albumin weakens immune defense mechanisms, resulting in a vicious circle associated with cancer. Oba et al. observed that DFS was significantly lower in the high ΔPNI group than in the low ΔPNI group (optimal cut-off value: 5.26, *P* = 0.015) when evaluating the prognostic impact of PNI changes in patients with breast cancer receiving NAC. These results suggest that maintaining nutritional status during NAC may lead to better treatment outcomes for patients with breast cancer (29). In our study,there is no significant difference was observed in pretreatment PNI between pCR and non-pCR groups. However, post-treatment PNI was greater in the pCR group than in the non-pCR group (*P* = 0.032). With regard to ΔPNI, the pCR group (**X **= −3.73) exhibited a smaller decrease compared to the non-pCR group (**X **= −5.08), although this difference was not statistically significant (*P* = 0.095). A potential explanation is that maintaining good nutritional and immune status during NAC may correlate with the curative effects.

As this was a retrospective single-center study, certain study limitations should be noted. First, the sample size was small, especially in the pCR group, which may have resulted in selection bias. Second, further multicenter studies are required for validation. Furthermore, there is a lack of continuous assessment of neoadjuvant treatment efficacy and lack of comparison with existing imaging methods for assessing neoadjuvant efficacy.

Further research on the relationship between chronic inflammation, inflammation-related parameters, and breast cancer is warranted to facilitate individualized treatment of breast cancer and prediction of efficacy.

## Conclusions

Pretreatment NLR, PLR, and SII can be used as predictors of pCR in patients with breast cancer undergoing NAC. Pretreatment SII has a higher predictive value, and patients with low SII are more likely to achieve pCR.

## Data Availability

The raw data supporting the conclusions of this article will be made available by the authors, without undue reservation.
